# Pre- and Postnatal Risk Factors in Relation to Allergic Rhinitis in School-Aged Children in China

**DOI:** 10.1371/journal.pone.0114022

**Published:** 2015-02-03

**Authors:** Youjin Li, Yanrui Jiang, Shenghui Li, Xiaoming Shen, Jinfen Liu, Fan Jiang

**Affiliations:** 1 Department of Otorhinolaryngology-Head & Neck Surgery, Shanghai Children's Medical Center affiliated with Shanghai Jiao Tong University School of Medicine, Shanghai, P. R. China; 2 MOE-Shanghai Key Laboratory of Children’s Environmental Health, Shanghai, P. R. China; 3 Department of Developmental and Behavioral Pediatrics, Shanghai Children's Medical Center affiliated with Shanghai Jiao Tong University School of Medicine, Shanghai, P. R. China; 4 Department of Pediatrics, Shanghai Children's Medical Center affiliated with Shanghai Jiao Tong University School of Medicine, Shanghai, P. R. China; 5 Shanghai Jiao Tong University Pediatric Institute, Shanghai, P. R. China; Beijing Institiute of Otolaryngology, CHINA

## Abstract

**Objective:**

The objective of this study was to investigate the relationship between prenatal and postnatal risk factors and the prevalence of allergic rhinitis (AR) in Chinese children of specific ages.

**Study Design:**

This study was a cross-sectional survey. Students from 8 metropolitan cities in China were studied in November and December, 2005. There were 20,803 elementary-school Chinese children (49.6% boys, mean age, 9.19 years) enrolled. Questions from the standard questionnaire of the International Study of Asthma and Allergies in Children were completed to enable us to examine the pattern of current AR. The potential confounders and pre-and postnatal risk factors were analyzed using logistic regression.

**Results:**

The overall prevalence of AR was found in this study to be 9.8%. After adjusting for several likely confounders, there was a higher likelihood of AR in school-aged children who were not exclusively breastfed in the first 4 months of their lives (odds ratio [OR]: 1.28; 95% confidence interval [CI]: 1.16–1.41), children who were born post-term (OR: 1.34; 95% CI: 1.12–1.60), children delivered by cesarean section (OR: 1.07; 95% CI: 1.00–1.19), or children born to mothers who experienced depressive symptoms during the pre- and postnatal periods (OR: 1.28; 95% CI: 1.15–1.42).

**Conclusions:**

AR in school-aged children was found to be associated with pre- and postnatal events. These findings indicate that strategies to reduce exposure to risk factors during pre- and postnatal periods for childhood allergies might be warranted.

## Introduction

Allergic rhinitis (AR), a chronic inflammatory disease of the upper airway, is not only one of the most common chronic diseases in children, but also a global health problem [1]. AR is characterized by the symptoms of nasal congestion, rhinorrhea, sneezing, and nasal itching. The prevalence of childhood AR varies widely, ranging from 1.4% to approaching 46%. Furthermore, the prevalence of AR appears to be increasing globally, including in China [2,3]. In 2013, Zhang et al. [3] reported that the prevalence of epidemiological AR (data were based on surveys using a questionnaire) was 48% in 3–5 years old in Beijing, China.

Although the etiology of AR is uncertain, there is evidence to suggest that an atopic phenotype might be programmed in-utero [4]. Interactions between the microbial environment and the developing immune system are of high interest in understanding the modern-day epidemic of immune diseases. The human immune system of allergen-specific responses is already evident at birth and increases with age. It begins to respond to ubiquitous environmental allergens at a very early age and sensitization to allergens can occur even in the fetal stage [5]. A few reports based on data obtained from the International Study of Asthma and Allergies in Childhood (ISAAC) showed that a family history of atopy, frequent respiratory tract infections, antibiotics given in the first year of life, cats at home in the first year of life, ever breast feeding, high socioeconomic status and cesarean delivery were important independent risk factors for AR [6,7]. However, few large population-based studies have evaluated AR in childhood in relation to pre- and postnatal risk factors in China.

A national survey on children’s sleep patterns was conducted in eight Chinese cities in 2005 [8]. Because this survey covered a range of childhood illnesses, including AR and other allergic diseases, it provided a valuable opportunity to study the national prevalence in China of children’s AR and the influence of pre- or postnatal factors on subsequent development of AR.

## Materials and Methods

### Study design

The study based on childhood sleep patterns was conducted between November and December 2005 in eight cities in China through a cluster-stratified selection. The surveys were performed in areas of northern, southern, and central China, comprising Hohhot (HO), Urumqi (UR), Harbin (HA), Shanghai (SH), Guangzhou (GZ), Xi’an (XA), Wuhan (WH) and Chengdu (CD). These eight cities were chosen because they have high levels of health resources and public awareness about childhood allergic diseases, and doctors generally follow the national standard for the clinical diagnosis of AR. These cities were divided into two groups for this study according to their gross domestic product (GDP) and health-system coverage [9]. Those with a higher GDP and health-system coverage (GDP>22,380,000 RMB and health-system coverage>49.33%) were categorized as city level I and comprised SH, GZ, WH, and CD. The remaining cities, XA, HA, HO and UR, were categorized as city level II (GDP<22,380,000 RMB and health-system coverage<49.33%).

The study population comprised 5- to 13-year-old children attending the first to sixth grades of primary school. 3–10 districts were randomly selected for each city and within each district, 1–2 elementary schools were chosen. Thirty districts and 42 schools in urban areas, 9 districts and 13 schools in suburban areas constituted the final sample.

Rhinitis and asthma are linked by epidemiological, pathological and physiological characteristics and by a common therapeutic approach. According to the concept of “one airway, one disease” for asthma and rhinitis [10], 716 asthmatic children with and without AR were included, 315of whom had AR with asthma. The final sample consisted of 20,803 children.

### Data Collection

Study design, enrollment, criteria for inclusion and procedures for data collection were described in detail in previous articles [11]. In brief, data were collected by the Shanghai Key Laboratory of Children’s Environmental Health in Shanghai, China. Each center has a national steering committee that includes highly experienced pediatricians and epidemiologists who used a uniform research protocol and provided formal training for the survey interviewers. Consent forms were signed by parents or guardians, and the purpose of the study was explained to principals and teachers at the target schools. The questionnaires were completed by a parent or guardian of the child who was eligible to participate in the study. The project was set up by the Ministry of Education of the People’s Republic of China. The ethical application of this study was approved by the Institutional Review Board of Xinhua Hospital affiliated with Shanghai Jiao Tong University.

### Assessment Questionnaires

**Atopic disease questionnaires.** Key questions were taken from the Chinese version of the ISAAC questionnaire, which exhibited good reliability and validity to examine the prevalence of current AR to compare with results in the literature [12]. The questions regarding AR and asthma were as follows: “Has your child had allergic rhinitis in the past 12 months?” (Yes or no); “Has your child had asthma in the past 12 months?” (Yes or no).

**Sociodemographic questionnaires.** The sociodemographic questionnaire surveyed for factors that might have had a significant impact on AR. These comprised the following variables: gender, age, gestational age (<37 weeks/ 37–41 weeks/≥42 weeks), method of delivery (vaginal delivery/cesarean section), exclusive breastfeeding in the first 4 months (yes/no), parental educational level (middle school and below/high school/college and above), family income (yuan/month/person), resident area per captia (m^2^), family numbers (<3 persons/3 persons/≥ persons), mother having depressive symptoms during pre- and postnatal period (yes/no).

### Statistical analysis

Statistical analysis was performed including the use of the mean and standard deviation (SD) for continuous variables, and percentages for categorical variables. Firstly, the strength of the relationship between potential risk factors and AR was evaluated using logistic regression analysis. Secondly, the pre- and postnatal factors after adjustment for risk factors using logistic regression analyses were used.

In Model 1, the independent risk factors for AR were adjusted for the child’s age and gender. Model 2 was further adjusted for socioeconomic factors: paternal and maternal education, household income, family size and family numbers and city. All statistical analysis used SPSS statistical software package for Windows version 17.0.1 (SPSS Inc., Chicago, IL, USA). Significance was defined as a *p*<0.05.

## Results

Of the 21,030 children from the six grades of the chosen schools, 20,803 (response rate: 99.0%) participated in the survey and returned completed questionnaires. Of the total sample, 49.6% were males and the mean age was 9.19 years (SD = 1.76). The vast majority (19,905; 90.88%) were from the Han ethnic group, with the remaining children (1,998; 9.12%) from ethnic minorities. The overall prevalence of AR was 9.8% in this population.

The prevalence rates of AR in children <7, 7, 8, 9, 10, 11, and ≥ 12 years were 9.1, 8.3, 10.2, 10.1, 10.1, 11.0 and 10.7%, respectively. There were statistically significant differences in the prevalence of AR among the different ages (OR: 1.05; 95% CI:1.02–1.07, *p* = 0.001).

As shown in Table 1, parental education level, household income per capita, the number of family members, and resident area per capita, were significantly associated with AR. Furthermore, children who were in cities with higher GDP and health-system coverage had a higher prevalence of AR than those with a lower GDP and health-system coverage. Potential correlates between pre-, postnatal risk factors and AR in school-aged children (Table 1) encompassed no exclusive breastfeeding in the first 4 months (OR: 1.50; 95% CI: 1.37–1.65, *p*<0.001), post-term birth or delivery by cesarean section (OR: 1.42; 95% CI: 1.20–1.69, *p*<0.001; OR: 1.36; 95% CI: 1.23–1.49, *p*<0.001), and mother exhibiting depressive symptoms during the pre- and postnatal periods (OR: 1.16; 95% CI: 1.05–1.29, *p* = 0.014). However, parental smoking, and/or parental alcohol abuse had no significant association with AR measurements.

**Table 1:** Relationship between potential risk factors and allergic rhinitis in schoolchildren (n = 2,047) in China (N = 20,803).

Potential risk factors		% (n/N)	Unadjusted OR(95% CI)	*p* value*
Pre- and postnatal factors of AR				
Exclusive breastfeeding in the first 4 months				
	Yes	8.5(1164/13616)	1	<0.001
	No	12.3(881/7152)	1.50(1.37–1.65)	
	Missing	5.7(2/35)	—	
Age of gestation (weeks)				<0.001
	term	9.6(1734/17981)	1	
	preterm	10.3(118/1151)	1.07(0.88–1.30)	
	Post-term	13.2(159/1207)	1.42(1.20–1.69)	
	Missing	7.8(36/464)	—	
Mode of delivery				<0.001
	vaginal	8.9(1243/13927)	1	
	cesarean	11.7(796/6786)	1.36(1.23–1.49)	
	Missing	8.9(8/90)	—	
Mother exhibiting depression during pre- and postnatal period				0.014
	No	9.5(1458/15309)	1	
	Yes	10.9(549/5042)	1.16(1.05–1.29)	
	Missing	8.8(40/452)	—	
Children’s characteristics				
Age continuous, (1 year units)		9.8(2047/20803)	1.05(1.02–1.07)	0.001
Gender				<0.001
	Boy	11.8(1215/10311)	1.55(1.41–1.70)	
	Girl	7.9(832/10492)	1	
City				<0.001
	I(SH, GZ,WH,CD)	12.4(1527/12279)	2.19(1.97–2.43)	
	II(XA, HA,HO,UR)	6.1(520/8524)	1	
Maternal education				<0.001
	middle school and below	6.4(373/5812)	1	
	high school	9.6(658/6841)	1.55(1.36–1.77)	
	College and above	12.6(984/7783)	2.11(1.86–2.39)	
	Missing	8.7 (32/367)	—	
Paternal education				<0.001
	high school and below	6.4 (318/4962)	1	
	high school	9.4(667/7089)	1.52(1.32–1.74)	
	college and above	12.2(1049/8633)	2.02(1.77–2.30)	
	Missing	10.9(13/119)	—	
Resident area per capita (m^2^)				<0.001
	<15	7.4(222/2991)	1	
	15–25	8.7(549/6324)	1.19(1.01–1.40)	
	25–35	10.5(548/5212)	1.47(1.25–1.73)	
	>35	11.9(707/5949)	1.68(1.44–1.97)	
	Missing	6.4(21/327)	—	
Household income per capita(RMB/month)				<0.001
	<800	5.7(221/3870)	1	
	800–1500	8(535/6727)	1.43(1.21–1.68)	
	1500–2500	10.5(513/4905)	1.93(1.64–2.27)	
	>2500	14.9(752/5061)	2.88(2.47–3.37)	
	Missing	10.8(26/240)	—	
Family numbers				0.004
	<3 persons	11(159/1450)	1.26(1.05–1.51)	
	3 persons	10.4(1157/11158)	1.18(1.07–1.30)	
	≥4 persons	8.9(725/8115)	1	
	Missing	7.5(6/80)	—	
Maternal alcohol abuse				0.55
	Yes	11(16/145)	1.14(0.68–1.92)	
	No	9.8(2030/20633)	1	
	Missing	4.0(1/25)	—	
Paternal alcohol abuse				0.66
	Yes	9.4(211/2240)	0.94(0.82–1.10)	
	No	9.9(1830/18487)	1	
	Missing	7.9(6/76)	—	
Maternal smoking				0.84
	Yes	10.6(46/433)	1.09(0.81–1.49)	
	No	9.8(1996/20324)	1	
	Missing	10.9(5/46)	—	
Paternal smoking				0.74
	Yes	9.9(1175/11810)	1.03(0.94–1.13)	
	No	9.7(864/8923)	1	
	Missing	11.4(8/70)	—	

n = number of observations with AR, N = total number of observations.

AR = allergic rhinitis, OR = odds ratio, CI = confidence interval, SH = Shanghai, GZ = Guangzhou, CD = Chengdu, XA = Xin’an, WH = Wuhan, HA = Harbin, HO = Hohhot, UR = Urumqi.

*p value from univariate logistic regression.

In multivariate logistic regression for Model 1 (Table 2), after adjusting for age, gender and city confounders, AR was related to pre- and postnatal risk factors such as no exclusive breastfeeding in the first 4 months, post-term birth, cesarean section, and the mother exhibiting depressive symptoms during the pre- and postnatal periods. In Model 2 (Table 2), the relationship between no exclusive breastfeeding and AR (odds ratio [OR]: 1.28; 95% CI: 1.16–1.41, *p*<0.001), post-term birth and AR (OR: 1.34; 95%CI: 1.12–1.60, *p* = 0.001), cesarean section and AR (adjusted OR: 1.07; 95% CI: 1.00–1.19, *p* = 0.040), remained statistically significant after adjusting for all confounders. Of the aforementioned pre- and postnatal risk factors, the other significant risk factor for AR was the mother exhibiting depressive symptoms during the pre- and postnatal periods (OR: 1.28; 95%CI, 1.15–1.42, *p*<0.001). In addition, interactions between the covariates in multivariate models showed no significant differences.

**Table 2:** Pre- and postnatal risk factors of allergic rhinitis in school children from multivariate logistic regression models.

Variable		Model1 Adjusted^1^ OR(95% CI)	Model2 Adjusted^2^ OR(95% CI)
Exclusive breastfeeding in the first 4 months			
	Yes	1.00	1.00
	No	1.39(1.26–1.52)	1.28(1.16–1.41)
Age of gestation (weeks)			
	Term	1.00	1.00
	Preterm	0.95(0.78–1.16)	0.99(0.81–1.21)
	Post-term	1.3(1.09–1.55)	1.34(1.12–1.60)
Mode of delivery			
	Vaginal	1.00	1.00
	Cesarean	1.24(1.12–1.36)	1.07(1.00–1.19)
Mother exhibiting symptoms of depression during pre- and postnatal period			
	No	1.00	1.00
	Yes	1.25(1.12–1.38)	1.28(1.15–1.42)

OR = odds ratio, CI = confidence interval. Values highlighted in bold indicate OR within 95% CI and statistically significant at *p*<0.05.

^1^Adjusted for age, gender, city.

^2^Adjustedfor age, gender, household income per capita, family numbers, resident area per capita, parental education level, city.

## Discussion

The national pattern of prevalence and associated pre- and postnatal risk factors of childhood allergies in China is available in this study. The overall prevalence of AR in Chinese school-aged children according to this study was 9.6%, and significantly higher rates of AR were observed in boys than in girls (OR:1.55; 95%CI:1.41–1.70). However, the prevalence of AR exhibited a different pattern from those of previously reported studies, peaking at the age of 11(11.0%), and not in younger school children [13]. The most recent report of Zhang et al. [3] found that the prevalence of epidemiological AR is 48% in Beijing, China, which is higher than that reported from other studies in China, which ranged from 7.83- to 42.2% [14–16]. The extremely wide range resulted from sampling strategy, the definition of AR, the ages of the children and the study population being from different regions. In addition, there were significant differences in our study results across cities with different GDP and health-system coverage [17]. Susceptibility to AR is considered to be dependent on a complex interplay of genetic factors, nonspecific adjuvant factors, and environmental exposure to allergens. We have demonstrated a strong direct association between AR in Chinese school-aged children and pre- and postnatal risk factors, such as no exclusive breastfeeding in the first 4 months, post-term birth, cesarean section, and the mother exhibiting depressive symptoms during the pre- and postnatal periods.

The same conclusions were drawn in a multidisciplinary review regarding the effect of breastfeeding on later development of allergic diseases [18]. The biological plausibility of the allergy-preventive effect of breastfeeding has been studied in a number of ways including traces of food proteins consumed by the mother conducive to tolerance of these foods [19] and reducing inflammatory responses by destroying microbes [20]. However, some studies have not supported that there is a protective effect of exclusive breastfeeding in the first 4 months on asthma or allergies [21]. Even though that, in China, exclusive breastfeeding has been shown to have protective effects against AR at school age. Nevertheless, China’s recently updated national rate of “exclusive breast feeding for 6 months” reached 21.6%, which, although still far below the World Health Organization target of 100% [22]. Therefore, breastfeeding is likely to be of importance in AR prevention, particularly in countries where the mother can arrange to breastfeed her baby for a substantial period of time.

In general, some authors argue that low gestational age is associated with a decreased risk of AR [23]. However, in this study we found a significantly higher prevalence of AR in school children born post-term, which is similar to the results of some studies that support the correlation between AR and a high gestational age or high birth weight [24]. Other studies, however, have failed to confirm these findings [25,26]. Recent immunological research has demonstrated that atopic conditions, including AR, are believed to reflect a predominantly T helper cell 2(Th2) lymphocyte response to environmental antigens [27]. Some studies [28]suggested that the maternal immune response shifts toward Th2 during a successful pregnancy because T helper cell 1(Th1) cytokines might be harmful to the maintenance of the pregnancy. One hypothesis could be that longer exposure to Th2 cytokines during a prolonged pregnancy could modify the differentiation of fetal Th precursors into Th2 cells and increase the risk of atopy later in life [28].

A recent WHO survey revealed that China had the world’s highest rate of cesarean sections (up to 46.2%), of which 25% were done without medical indications, far exceeding the upper threshold of 15% recommended by WHO [29]. Similarly, there was a fairly high prevalence of cesarean sections in our study (32.6%), but our study demonstrated that cesarean section was independently associated with AR in school children after adjusting for socioeconomic factors. These results are consistent with recently published studies [30]. A comparison of the effects of cesarean delivery with those of vaginal delivery should also be recognized, such as the absence of vaginal compression of the baby’s chest and/or decreased stress and labor during cesarean section. However, necessary stress and labor during vaginal delivery induce a catecholamine surge [31], and high levels of cortisol [32], and pulmonary surfactant [33], all of which contribute considerably to normal postnatal lung adaptation and development. It has also been hypothesized that cesarean section resulting in exposure to maternal microflora might differ or reduce the maturation of babies’ immune systems [34]. Such changes might influence the balance between Th1 and Th2 lymphocytes early in life [35]. An important controlling factor of contact with micro-organisms forms the basis of what has become known as the “hygiene hypothesis” of allergic disease, which attempts to explain the possible association between exposure to infections, such as during cesarean delivery, and the development of allergic disorders. Therefore, the decision of whether to perform or consent to a cesarean-section delivery needs to be made with caution.

Another new finding in this study was the positive relationship between depressive symptoms in mothers during the pre- and postnatal periods and subsequent AR in the school-age offspring. However, it is unclear whether there are any critical periods of exposure. The association of AR with pre- and postnatal exposure to maternal depressive symptoms has not been reported in human studies, but some explanation has been provided. Maternal stress associated with sustained excessive cortisol secretion could affect the developing immune system, especially Th1/Th2 cell balance of the child [36]. Altering the regulation of the hypothalamic-pituitary-adrenal axis has been suggested as a plausible pathway for reported associations between parental stress and asthma or wheezing in children [37]. We also suggest that there is a negative association between prenatal stressors and their impact on immune response in the offspring.

We acknowledge that there are some limitations to our study. Firstly, there was a lack of a more-expanded panel of questions regarding family history of atopic diseases because this study was originally designed to address the issue of childhood sleep patterns. In a published meta-analysis study [38], data showed that exclusive breastfeeding during the first 3 months after birth protects against AR in children, both with and without a family history of atopy. There was still an increased risk of developing AR in children delivered by cesarean section despite adjusting for confounders such as the mother’s use of allergy medications as a proxy for maternal atopy (OR: 1.37; 95% CI: 1.14–1.63). These findings suggest that both breastfeeding and cesarean section independent of a parental history of allergies were associated with a decreased risk of AR in children [39]. However recently, Kolokotroni et al. [40] reported that birth by cesarean section was associated with asthma and atopic sensitization, which appears more pronounced in children with a family history of allergies. It would be valuable to conduct future studies to clarify these contradictory findings. The second limitation was the reliance on parent-reported data on children’s nasal symptoms and behaviors without objective confirmation by a physician, which might create a rater bias and inaccuracy of the results. Finally, the present study was cross-sectional and retrospective; therefore, the results should be further confirmed through an objective observational or prospective study.

The most important strength of this study was its ability to examine the association between pre-, postnatal risk factors and AR in a large cross-sectional survey with high representation. To our knowledge, this is the first report to provide information about breastfeeding, age of gestation, mode of delivery and mother exhibiting signs of depression during the pre- and postnatal periods between risk factors and AR in Chinese school-aged children.

## Conclusions

In this study, we provide evidence of how the pre- and postnatal risk factors such as post-term birth, cesarean section, no exclusive breastfeeding in the first 4 months, and the mother exhibiting depressive symptoms during the pre- and postnatal periods, might be related to the prevalence of AR in children. There is a need for worldwide research to further explore these factors and to develop policies for early intervention in a child’s life to prevent AR. Meanwhile, additional research will most likely ensue to confirm the critical exposure period and the underlying immunologic mechanisms.
